# Blood microbiome signatures in systemic diseases: current insights, methodological pitfalls, and future horizons

**DOI:** 10.3389/fcimb.2025.1616029

**Published:** 2025-07-28

**Authors:** Ikram Khan, Muhammad Irfan, Imran Khan, Uzma Noor, Xiaodong Xie, Zhiqiang Li

**Affiliations:** ^1^ Department of Genetics, School of Basic Medical Sciences, Lanzhou University, Lanzhou, Gansu, China; ^2^ Department of Medical Laboratory Technology, Xcito School of Nursing and Allied Health Sciences, Chakdara, Khyber Pakhtunkhwa, Pakistan; ^3^ Department of Microecology, School of Basic Medical Sciences, Dalian Medical University, Dalian, Liaoning, China; ^4^ Department of Physiology, College of Basic Medical Sciences, Dalian Medical University, Dalian, Liaoning, China; ^5^ School of Stomatology, Key Laboratory of Oral Disease, Northwest Minzu University, Lanzhou, Gansu, China

**Keywords:** systemic diseases, blood microbiome, controversies, challenges, future directions

## Abstract

The human-associated microbiome, encompassing diverse microbial communities across body sites, plays a pivotal role in maintaining host homeostasis. Disruption of this balance, termed dysbiosis, has been implicated in a spectrum of pathophysiological conditions. Traditionally, blood was considered a sterile microenvironment. However, emerging insights into the blood microbiome challenge the paradigm of blood sterility, revealing microbial signatures, including cell-free DNA and viable taxa, with putative implications for host physiology and disease. The blood taxonomic profile at the phylum level is dominated by Proteobacteria, with Bacteroidetes, Actinobacteria, and Firmicutes following in abundance. Dysbiosis in blood microbiome composition may indicate or contribute to systemic dysregulation, pointing to its potential role in disease etiology. These findings highlight the blood microbiome as a possible driver in the pathogenesis of infectious and non-infectious diseases, neurodegenerative disorders, and immune-mediated conditions. The detection of specific microbial profiles in circulation holds promise for biomarker discovery, enhancing disease stratification, and informing precision therapeutic strategies. However, advancing this field requires overcoming methodological challenges, including contamination control, standardization, and reproducibility. This review aims to present blood microbiome biomarkers across infectious, non-infectious, neurodegenerative, and immune-mediated diseases, while critically examining methodological variations, controversies, limitations, and future research directions. Elucidating these factors is critical to advancing blood microbiome biomarker validation and therapeutic targeting, thereby refining mechanistic insights into systemic disease pathogenesis.

## Introduction

The human microbiome, an intricate assemblage of microorganisms residing within and on the human body, constitutes a dynamic ecosystem that profoundly influences human health. The composition and diversity of this microbiome have been linked to numerous physiological processes and disease states (Heintz-Buschart and Wilmes, 2018). Dysbiosis, characterized by an imbalance or perturbation in the microbiome’s equilibrium, has garnered attention for its potential implications in a wide array of health disorders (Shukla et al., 2024). Dysbiosis in the gut microbiome plays a potential role in both human health and disease states (Chen et al., 2021); however, the association between gut microbiome and human diseases is overlooked (Velmurugan et al., 2020). Beyond gut microbiome dysbiosis, recent metagenomic analyses have renewed interest in the long-standing hypothesis of a blood-resident microbiome, underscoring its potential role in disease pathophysiology (Tedeschi et al., 1969; Khan et al., 2022a). These developments compel further scrutiny into the nature and significance of microbes in the bloodstream.

The circulation is a closed system, and the blood in healthy individuals was earlier believed to represent a sterile environment, which is the basis for safe blood transfusions (Damgaard et al., 2015). However, recent studies have challenged this concept, revealing the presence of resident microbiomes in both healthy individuals and those with diseases (Castillo et al., 2019; Whittle et al., 2019; Velmurugan et al., 2020). These studies confirmed the presence of live bacteria, bacterial DNA (and associated metabolites), viral DNA (e.g., Rhabdoviridae and Anelloviridae), archaeal DNA (e.g., Euryarchaeota), and fungi (e.g., Basidiomycota, Ascomycota) in the blood (Panaiotov et al., 2018). While these findings support the idea of a circulating microbiome, debate persists about whether these microbes represent a stable, endogenous community or are transient migrants from colonized body sites (Jagare et al., 2023). For example, a recent large-scale study reported no consistent core blood microbiome, reinforcing the hypothesis of peripheral origin through translocation (Tan et al., 2023). These conflicting results underscore the complexity and need for standardization in blood microbiome research.

Nonetheless, a mountain of evidence suggests that the blood microbiome plays a crucial role in the development of various human diseases, including diabetes mellitus (Qiu et al., 2019), allergies (Funkhouser and Bordenstein, 2013), asthma (Lee et al., 2020), irritable bowel syndrome (Jagare et al., 2023), cardiovascular diseases (CVDs) (Amar et al., 2019; Khan et al., 2022b, 2022), and cancer (Poore et al., 2020; Søby et al., 2020; Yang et al., 2021). The blood microbiomes have also been detected in animals such as cats (Vientoos-Plotts et al., 2017), dogs (Scarsella et al., 2020, 2023), cows (Scarsella et al., 2021), pigs (Hyun et al., 2021), goats (Tilahun et al., 2022), and camels (Mohamed et al., 2021) in both healthy and diseased states. However, it remains unclear whether blood constitutes a stable ecological niche for bacteria with functional roles in human physiology or merely serves as a transient conduit for microbial migration between colonized sites. The most likely source of blood-associated microbes is translocation from microbe-rich environments, particularly the gastrointestinal tract and oral cavity, often triggered by mucosal injury (e.g., tooth brushing) or increased intestinal permeability (Castillo et al., 2019; Velmurugan et al., 2020). These observations lay the foundation for examining how blood microbiome signatures intersect with systemic disease processes.

Thus, this review examines the emerging concept of the blood microbiome and its potential involvement in the pathogenesis of systemic diseases. Drawing on recent findings that challenge the long-standing notion of blood sterility, we explore microbial signatures, both cell-free and viable, detected in circulation and their associations with infectious, non-infectious, neurodegenerative, and immune-mediated conditions. We emphasize the diagnostic and prognostic promise of blood microbiome profiles while addressing critical methodological pitfalls, including contamination risks and lack of standardization in low-biomass microbiome studies. By identifying key controversies and outlining future research priorities, this review aims to advance the clinical and mechanistic understanding of the blood microbiome in systemic disease.

## Search strategy and eligibility criteria

We performed a systematic search of English-language literature via PubMed, Web of Science, and Google Scholar using MeSH terms and keywords including blood bacteria, blood microbiome, blood microbiota, circulating bacteria, circulating microbiome, circulating microbiota, bacteremia, and transient bacteremia. The search targeted cohort studies employing blood, plasma, or serum microbiome analyses using methods such as 16S rRNA sequencing, high-throughput RNA sequencing, Illumina MiSeq, shotgun metagenomic sequencing of cell-free DNA, and pyrosequencing. Eligible studies were observational (cohort, case-control, or retrospective) in patients with infectious, noninfectious, neurodegenerative, or immune-mediated conditions, analyzing blood-derived samples. Exclusion criteria included animal studies, intervention trials involving prebiotics or probiotics, studies assessing skin or gut microbiota via blood, those without comparative data on systemic diseases, and studies focusing on cardiometabolic or unrelated conditions.

## Blood microbiome

The human microbiome is a diverse and dynamic community of microorganisms, including bacteria, viruses, fungi, and archaea, that inhabit various sites within the body. These microbes form a complex ecosystem, interacting closely and symbiotically with the human host. Understanding the composition and dynamics of the blood microbiome is essential for uncovering its specific impact on human health and disease [17–20], as maintaining balance within these microbial communities is critical for overall host function and resilience. **Table 1** summarizes the key components of the microbiome, highlighting the unique characteristics and predominant taxa of the blood microbial community.

**Table 1:** Key blood microbial taxa and their possible roles in immune modulation, metabolism, and host homeostasis under normal conditions.

Microbiome	Species	Mechanism of action	Ref.
Bacteria	Proteobacteria, Bacteroidetes, Actinobacteria, and Firmicutes	Proteobacteria: Often involved in maintaining immune homeostasis by interacting with host pattern recognition receptors (e.g., TLRs) through their surface molecules like lipopolysaccharides (LPS), which can modulate immune signaling.; Bacteroidetes: Contribute to the metabolism of complex carbohydrates and production of short-chain fatty acids (SCFAs), which regulate host immune responses and gut barrier integrity.; Actinobacteria: Participate in maintaining skin and mucosal barrier functions; some species produce antimicrobial compounds that inhibit pathogenic bacteria and modulate local immune responses.; Firmicutes: Play a key role in fermenting dietary fibers into SCFAs (like butyrate), supporting energy metabolism, anti-inflammatory effects, and epithelial cell health.	(Païssé et al., 2016; Shah et al., 2019; Whittle et al., 2019)
Viruses	Rhabdoviridae and Anelloviridae	Rhabdoviridae: Typically infects host cells by attaching to cell surface receptors, entering via endocytosis, and replicating in the cytoplasm. While not usually persistent in blood, viral components can trigger innate immune responses by activating pattern recognition receptors (PRRs), such as RIG-I-like receptors, leading to interferon production and antiviral defenses.; Anelloviridae: These viruses establish chronic, mostly asymptomatic infections with persistent low-level replication in blood cells or tissues. Their DNA can modulate the host immune system subtly, often avoiding strong immune activation but potentially influencing immune homeostasis and inflammatory status by interacting with immune cells or altering cytokine profiles.	(Castillo et al., 2019; Liang et al., 2021)
Fungi	Basidiomycota and Ascomycota	Basidiomycota and Ascomycota fungi release cell wall components such as β-glucans, mannans, and chitin into the bloodstream. These molecules are recognized by pattern recognition receptors (PRRs) like Dectin-1, Toll-like receptors (TLRs), and the complement system on immune cells.This recognition activates innate immune responses, triggering cytokine release (e.g., TNF-α, IL-6) and recruitment of neutrophils and macrophages to maintain immune surveillance and prevent fungal overgrowth. In healthy individuals, these low-level fungal signatures may contribute to immune system “training” and modulation without causing infection, supporting homeostasis through controlled immune activation.	(Panaiotov et al., 2018)
Archaea	Euryarchaeota	Archaeal DNA from Euryarchaeota detected in blood may influence host physiology primarily through interactions with the immune system. Though direct pathogenic roles are unclear, archaeal components such as methanogenic enzymes can modulate local microbial communities and immune responses by influencing inflammatory signaling pathways and contributing to redox balance, thus potentially affecting systemic immune homeostasis under normal conditions.	(Dinakaran et al., 2014)

## Alterations in the blood microbiome in systemic diseases

Although the presence of a blood microbiome was first noted over five decades ago, it has gained significant scientific interest since 2001 (Nikkari et al., 2001), with mounting evidence connecting it to various human diseases. Recent research has increasingly focused on the complex dynamics of the blood microbiome and its potential role in disease pathogenesis. This section synthesizes current knowledge on the composition and diversity of the blood microbiome across four key categories: infectious diseases, non-infectious diseases, neurological disorders, and immune-mediated conditions (**Table 2**).

**Table 2:** Blood microbiome composition and diversity across infectious and non-infectious diseases, neurodegenerative disorders, and immune-mediated conditions.

Participants	Biomaterial	Method	Key findings	Ref.
242 HIV patients	Blood	qPCR targeted specific 16S rDNA regions	Patients with HIV infection who were untreated had the greatest 16S rDNA copy number. Both the bacterial 16S rDNA and the HIV viral load were linked with circulating LPS. Increased 16S rDNA in HIV patients is linked to slower CD4 T cell recovery.	(Jiang et al., 2009)
17 Healthy, 13 with ascites and 14 without ascites patients	Blood	V4 region 16S rDNA	A higher level of Order Clostridiales was observed in ascites patients, with a declined level of family Moraxellaceae.	(Santiago et al., 2016)
57 Healthy and 58 Parkinson’s disease patients	Blood	V3-V4 regions of 16S rDNA	Positive correlations were observed between elevated levels of *Amaricoccus, Bosea, Janthinobacterium, Nesterenkonia*, and *Sphingobacterium* and Hamilton Anxiety Scale scores in Parkinson’s patients, while higher levels of *Aquabacterium, Bdellovibrio*, and *Leucobacter* showed positive correlations with Hamilton Depression Scale scores.	(Qian et al., 2018)
26 Healthy and 27 Hidradenitis Suppurativa patients	Blood	V3-V4 regions of 16S rDNA	There were no differences observed between the blood types of skin disease patients and healthy individuals.	(Ring et al., 2018)
49 Healthy, 48 schizophrenia, 47 amyotrophic lateral sclerosis, and 48 bipolar disorder	Whole blood	High-quality unmapped RNA sequencing	Proteobacteria, Firmicutes, and Cyanobacteria were the three dominannt phyla in both groups, while patients with schizophrenia had higher levels of microbial diversity.	(Olde Loohuis et al., 2018)
5 Healthy and 5 Asthma patients	Plasma	V4 region 16S rDNA	Both groups exhibited the presence of Proteobacteria, Actinobacteria, Firmicutes, and Bacteroidetes in their blood.	(Whittle et al., 2019)
30 Healthy and 10 Rosacea patients	Blood	V3-V4 regions of 16S rDNA	Rosacea patients showed elevated levels of Chromatiaceae and Fusobacteriaceae families. And *Rheinheimera, Sphingobium, Paracoccus*, and *Marinobacter* genera.	(Yun et al., 2019)
14 Healthy and 66 liver Cirrhosis patients	Blood	V3-V4 regions of 16S rDNA	Cirrhosis patients showed elevated levels of Enterobacteriaceae and reduced levels of *Akkermansia*, Rikenellaceae, and Erysipelotrichales. While both cirrhosis and hepatocellular carcinoma demonstrate elevated levels of Enterobacteriaceae and *Bacteroides*, along with reduced *Bifidobacterium*.	(Kajihara et al., 2019)
7 Liver cirrhosis patients	Blood	16S rDNA sequencing	*Staphylococcus* and *Acinetobacter* were successfully cultivated, and the amount of the blood microbiome strongly linked with inflammatory cytokines.	(Schierwagen et al., 2019)
260 Healthy and 190 Asthma patients	Blood	V3-V4 regions of 16S rDNA	A higher rate of eosinophilic asthma was associated with *Escherichia/Shigella*. Mixed granulocytic asthma was connected to C*omamonas*.	(Lee et al., 2020)
30 Healthy and 19 women with SLE	Blood	V4 region 16S rDNA	Plasma autoantibody levels showed a positive correlation with the majority of the enriched microorganisms. Moreover, in PBMC culture, monocytes producing TNF-, IL-1, and IL-6 were stimulated by the heat-inactivated bacteria *Planococcus*.	(Alekseyenko et al., 2021)
56 Healthy and 56 Depression patients	Blood	V3-V4 regions of 16S rDNA	Patients showed heightened levels of *Kocuria, Chryseobacterium, Parvimonas*, and *Janthinobacterium*. Post-antidepressant treatment, *Neisseria* and *Janthinobacterium* levels reverted to normal. Favorable antidepressant responses were associated with elevated Firmicute concentrations, reduced *Bosea* and *Tetrasphaera* abundance, and elevated plasma tryptophan levels. Treatment outcomes were linked to bacterial xenobiotics, amino acids, lipid, and carbohydrate metabolism.	(Ciocan et al., 2021)
8 Healthy and 20 Psoriasis patients	Blood	16S rDNA sequencing	Elevated blood microbes were associated with tryptophan metabolism, lipid biosynthesis, fatty acid metabolism, melanogenesis, as well as PPAR and adipokine signaling.	(Chang et al., 2021)
46 Healthy and 58 Cirrhosis patients	Blood	V1-V2 regions of 16S rDNA	The prevalence of Prevotella and *Escherichia/Shigella* was correlated with IL-8 concentrations in the hepatic vein.	(Gedgaudas et al., 2022)
9 Healthy an 11 SLE patients	Blood	V4 region 16S rDNA	SLE patients displayed distinct plasma and gut microbial profiles, with an observed enrichment of the phylum Gemmatimonadetes in their plasma.	(James et al., 2022)
32 HCV-induced portal hypertension	Blood	V3-V4 regions of 16S rDNA	15 patients had better portal hypertension after receiving antiviral therapy. Fewer respondents had the genus *Massilia* and more of the order Corynebacteriales. Levels of IFN-, IL-17A, and TNF- were inversely linked with Corynebacteriales. Glycerol and lauric acid were linked to *Massilia*.	(Virseda-Berdices et al., 2022)
26 Healthyand 23 Irritable bowel syndrome patients	Blood	Metatranscriptome	The dominant genera in the blood microbiome included *Staphylococcus, Pseudomonas, Micrococcus, Delftia, Escherichia, Stutzerimonas, Ralstonia, Bradyrhizobium, Cutibacterium*, and *Mediterraneibacter.*	(Jagare et al., 2023)
24 Healthy, 30 treatment-naïve individuals, 31 immunological non-responders, and 30 immunological responders.	Blood and stool	Metagenomic sequencing	Positive correlations were observed between blood microbes like *P.* sp. *CAG:5226, E.* sp. *CAG:251, P. succinatutens, A. hallii, P.* sp. *AM34-19LB, P. plebeius*, and *P. gingivalis* with pro-inflammatory proteins and HIV DNA and RNA, while displaying a negative correlation with anti-inflammatory proteins, CD4+ T-cells, and the CD4/CD8 ratio. In contrast, *B. multivorans, B. thuringiensis, V. vulnificus*, and *A. baumannii* exhibited the opposite trend.	(Guo et al., 2023)

Systemic lupus erythematosus (SLE); human immune deficiency virus (HIV); Acquired immunodeficiency syndrome (AIDS); Lipopolysaccharide (LPS); Hidradenitis Suppurativa (HS); Diagnostic and Statistical Manual of Mental Disorders (DSM); Body mass index (BMI); Glomerular filtration rate (GFR); Major depressive disorder (MDD); Text Revision (TR); Antiretroviral therapy (ART); Spontaneous bacterial peritonitis (SBP); Hepatic venous pressure gradient (HVPG); Direct-acting antiviral (DAA); Sustained viral response (SVR); Interferons (IFNs); Tumor necrosis factor (TNF); Interleukin (IL).

## Alterations in the blood microbiome in infectious diseases

Despite advancements in molecular biology, genetics, computation, and medicinal chemistry, infectious diseases remain a major and persistent threat to public health. Addressing the challenges of pathogen outbreaks, pandemics, and antimicrobial resistance requires collaborative, interdisciplinary efforts. Integrating systems and synthetic biology with blood microbiome research can accelerate progress in understanding human health and disease.

Recent studies observed alterations in the blood microbiome among patients with Human Immunodeficiency Virus (HIV) infection (Luo et al., 2019; Ancona et al., 2021). Libertucci et al. observed increased levels of Proteobacteria and decreased levels of Actinobacteria and Firmicutes phyla in the blood of HIV-positive individuals. They also found that elevated levels of Staphylococcaceae could alter the blood microbiome due to combination antiviral therapy (cART) (Libertucci and Young, 2019). This is clinically relevant, as cART-treated individuals may develop autoreactive B-cells and autoantibodies, suggesting a potential link between Staphylococcus and autoimmune manifestations in HIV. Blood microbiome disturbances may arise from gut bacterial translocation triggered by mucosal immune dysfunction and consequent epithelial barrier damage. Despite the effectiveness of cART, which may include treatments like non-nucleoside reverse transcriptase inhibitors or protease inhibitors, the compromise of gut epithelial barriers may persist in individuals with HIV infection (Lee et al., 2020). Nevertheless, these treatments may still contribute to ongoing gut bacterial translocation and sustained damage to the gut barrier (Søby et al., 2020). Luo et al. identified the presence of Massilia and Haemophilus in the blood of HIV patients undergoing effective cART. This finding suggests that these microbes may trigger the release of proinflammatory cytokines in the peripheral blood microbiome, potentially contributing to the progression of chronic systemic inflammation over time (Alekseyenko et al., 2021). A recent study by Guo et al. identified a specific blood microbiome signature, including *P.* sp. *CAG:5226*, *E.* sp. *CAG:251*, *P. succinates*, *A. hallii*, *P.* sp. *AM34-19LB*, *P. plebeius*, and *P. gingivalis*, which exhibited positive correlations with pro-inflammatory proteins, HIV DNA, and RNA. In contrast, these microbes showed negative correlations with anti-inflammatory proteins, CD4+ T-cells, and the CD4/CD8 ratio. Additionally, microbes such as *B. multivorans*, *B. thuringiensis*, *V. vulnificus*, and *A. baumannii* demonstrated an opposing pattern, suggesting the potential for identifying effective microbial and immunotherapeutic strategies for managing HIV infection (Guo et al., 2023). These findings imply that antiretroviral therapy may impair intestinal barrier integrity, while HIV infection itself could modulate the blood microbiome.

**Figure 1:**
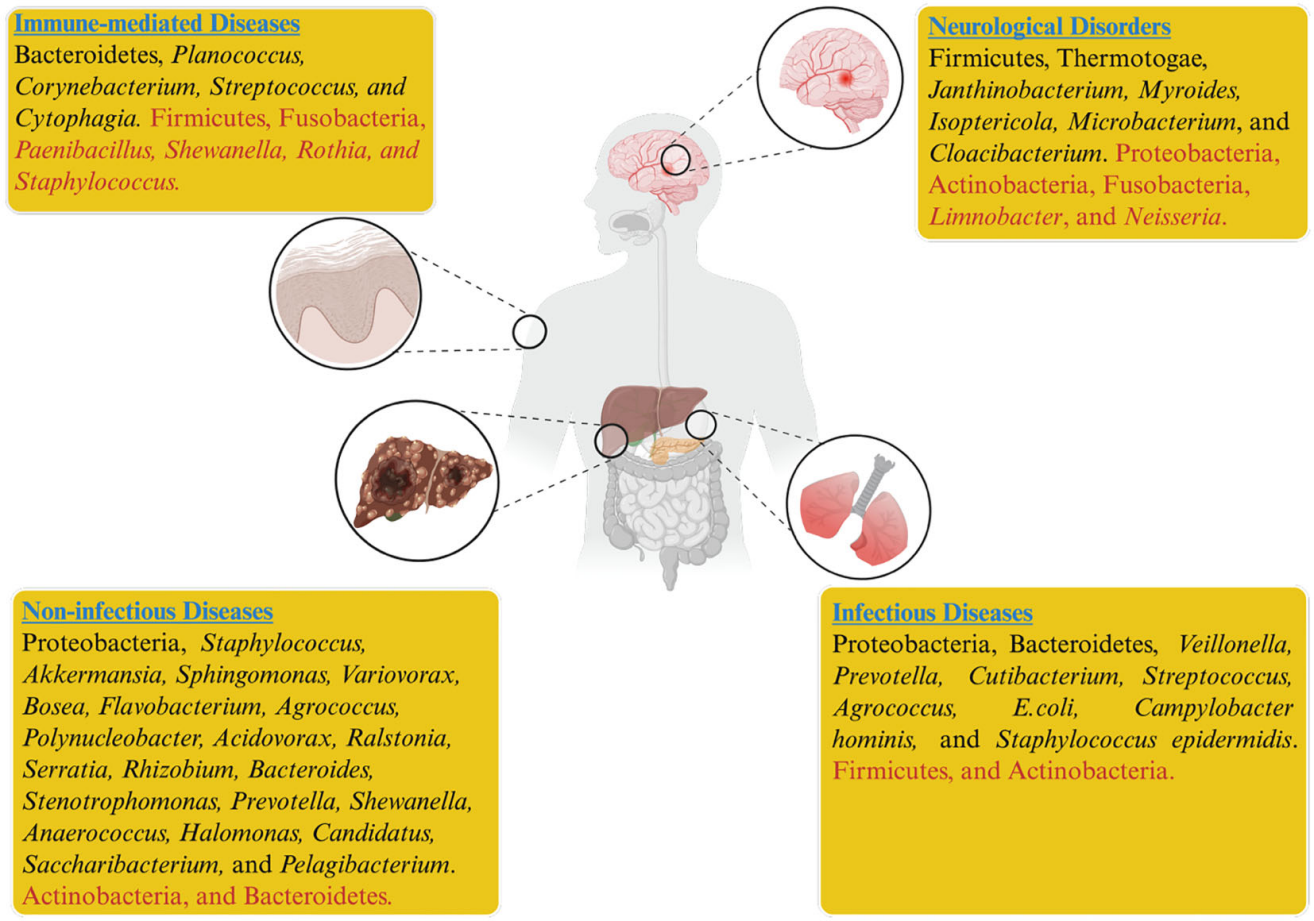
Dysbiosis of the blood microbiome has been associated with infectious, non-infectious, neurological, immune, and systemic diseases. In the figure, taxa shown in black indicate higher relative abundance, while those in red indicate reduced abundance across different disease states.

Evidence from the literature indicates that blood dysbiosis in septic patients is primarily associated with an overrepresentation of Proteobacteria or Bacteroidetes, while Actinobacteria are less commonly present. However, higher levels of *Agrococcus* within the Actinobacteria phylum have been suggested as a potential factor in the onset of sepsis (Païssé et al., 2016). Gosiewski et al. identified increased Proteobacteria and Bifidobacteriales in post-surgical sepsis patients, while Actinobacteria levels declined compared to healthy people (Gosiewski and Huminska, 2017). Several studies on lung diseases with suspected infections have identified significantly elevated levels of five bacterial genera in the blood microbiome, namely *Veillonella*, *Prevotella*, *Cutibacterium*, *Corynebacterium*, and *Streptococcus*, in patients with sarcoidosis (Hodzhev, 2023; Hodzhev et al., 2023). Investigating the blood microbiome in granulomatous lung diseases like sarcoidosis could provide insights into their origins and pathogenesis. The severity of COVID-19 has been associated with increased abundances of *E. coli*, *Bacillus* sp., *Campylobacter hominis*, *Pseudomonas* sp., *Thermoanaerobacter pseudethanolicus*, *Thermoanaerobacterium thermosaccharolyticum*, and *Staphylococcus epidermidis* (Dereschuk et al., 2021). These bacteria show that inflammation and the adaptive immune system are overactive (**Figure 1**). The findings provide insights into the blood microbiome profiles of smokers and COVID-19 patients, while also presenting a novel framework for investigating host–microbe interactions.

## Alterations in the blood microbiome in non-infectious diseases

Non-infectious diseases are a leading cause of mortality and morbidity worldwide. The biological effects of conditions such as diabetes, elevated total cholesterol, obesity, and smoking, along with behavioral risk factors like sedentary behavior, harmful alcohol consumption, and poor diet, resemble those associated with other non-infectious diseases like cancer, CVDs, and chronic respiratory diseases. Since a healthy lifestyle can mitigate disease risk, these factors are modifiable. In contrast, age, sex, race, and genetic background are non-modifiable risk factors that are also known to contribute to various non-infectious disorders (Finland, 2021). Despite traditional risk factors, the role of the blood microbiome in human health and its association with disease is an emerging focus of current research. The Lelouvier team identified a distinct link between liver fibrosis and an enrichment of Proteobacteria, particularly elevated levels of the genera *Sphingomonas*, *Variovorax*, and *Bosea* in patients with non-alcoholic fatty liver disease (Lelouvier et al., 2016). This finding underscores the potential role of microbial dysbiosis in the progression of liver diseases, suggesting that alterations in specific bacterial taxa may influence the pathophysiology of liver fibrosis and related conditions (**Figure 1**). Similarly, increased microbial diversity of 16S rDNA was observed in patients diagnosed with pancreatitis and schizophrenia (Li et al., 2018; Olde Loohuis et al., 2018). Li and colleagues revealed that patients with pancreatitis exhibited elevated levels of Bacteroidetes and decreased Actinobacteria. At the same time, specific genera, including *Serratia*, *Rhizobium*, *Bacteroides*, *Stenotrophomonas*, *Staphylococcus*, and *Prevotella*, were found in the patient’s blood, regardless of disease severity (Li et al., 2018). Schierwagen et al. reported that liver cirrhosis patients exhibited elevated levels of Acinetobacteria and *Staphylococcus*, which correlated with inflammatory cytokines, while beneficial taxa such as *Akkermansia*, *Rikenellaceae*, and *Erysipelotrichales* were reduced; notably, *Enterobacteriaceae* dominated, indicating a disease-related microbial shift (Schierwagen et al., 2019).

Furthermore, elevated levels of *Shewanella*, *Anaerococcus*, *Halomonas*, *Lachnospiraceae*, *Candidatus Saccharibacteria*, *Pelagibacterium*, and *Hyphomicrobiaceae*, along with decreased levels of *Bacteroidetes*, may contribute to the pathogenesis of rheumatoid arthritis (RA) (Hammad et al., 2020; Mo et al., 2020). Multiple other disorders have been linked to blood microbiome dysbiosis, such as Rosacea and psoriasis, two chronic dermatological conditions exhibiting distinct blood microbiome signatures (Yun et al., 2019). Moreover, Chang et al. found that the elevated levels of *Ralstonia, Staphylococcus*, and *Sphingomonas* in psoriasis suggest potential involvement in adipocytokine signaling and lipid metabolic pathways contributing to chronic inflammation (Chang et al., 2021). Contrarily, patients with hidradenitis suppurativa, another chronic inflammatory skin condition, exhibit a blood microbiome akin to that of healthy people, hinting that its pathophysiology may not be associated with bacteremia (Ring et al., 2018). Markova et al. investigated blood samples from mothers and children with autism, which were used to isolate fungi and bacteria in their L-form. While they suggested pathophysiology remains a hypothesis and the study lacked statistical analysis, it hints at a potential vertical transfer of pathogens from mother to child that could influence autism development (Markova, 2020). Wang et al. identified increased levels of *Flavobacterium, Agrococcus, Polynucleobacter*, and *Acidovorax* in the blood samples of surgical patients who subsequently experienced postoperative septic shock. These genera were significantly correlated with disease severity and organ failure assessment scores (Wang, 2021). In contrast, catheter insertion appears to elevate the abundance of Burkholderiales in the blood of mice receiving enteral nutrition (Lucchinetti et al., 2022). However, total parenteral nutrition leads to substantial changes in gut bacterial composition while having a relatively minor effect on the blood microbiome. A study by Simões-Silva demonstrated that bloodstream bacteria primarily stem from the dysbiotic gut microbiome in end-stage renal disease (ESRD), and hemodialysis, to some extent, exacerbates microinflammation by promoting gut microbiota translocation due to impaired gut barrier function (Simoes-Silva et al., 2018). Simões-Silva and colleagues further compared the peritoneal bacterial profile with other body parts. Although the blood microbiome profile exhibited the closest match to peritoneal bacteria, their findings confirmed significant differences between the peritoneal and blood microbiomes (Simões-Silva et al., 2020). Larger cohort studies are crucial to validate the role of the blood microbiome in the progression of non-infectious diseases, providing deeper insights into its potential as a biomarker for disease development and progression.

## Alterations in the blood microbiome in neurological diseases

The significant environmental and lifestyle changes in the modern era present a serious threat to human health, with the rise of various neurological disorders emerging as a major global challenge. Growing evidence suggests that the gut microbiota may influence brain function through the mediation of signaling pathways by microbial metabolites (Grochowska et al., 2019; Iannone et al., 2019). At the intersection of neuroscience and microbiology, groundbreaking studies from the past decade have revealed dynamic relationships between animals and their internal microbial populations. These interactions actively contribute to the development and functioning of neurological systems. The complex interplay of immunological, neural, and chemical signals plays a crucial role in maintaining health and advancing our understanding of neurological disorders (Morais et al., 2021). The gut-brain axis concept demonstrates how the gut microbiome can impact various brain-related health concerns (Mayer et al., 2022). Microbial components such as LPS and bacterial amyloid curli disseminate from the gut to the brain via circulation. These components tend to degrade the blood-brain barrier and cause aberrant accumulation of protein in the brain, which can lead to neuroinflammation (Suparan et al., 2022). Olde et al. analyzed the blood microbiome in patients with Amyotrophic Lateral Sclerosis (ALS), bipolar disorder, and schizophrenia. They observed elevated levels of *Planctomycetes* and *Thermotogae* in ALS patients compared to controls. However, patients with bipolar disorder and ALS displayed blood microbiomes that were similar to those of healthy individuals (Olde Loohuis et al., 2018). A previous study compared microbial DNA data derived from healthy individuals to human microbiome project (HMP) microbiome data. They demonstrated that, whereas the blood-microbiome closely resembles the skin and oral microbiomes, it differs substantially from the intestinal microbiome (Whittle et al., 2019). While most studies tend to consider the diffusion of bacteria into the blood-circulatory system as exceptional, this phenomenon may therefore occur rather frequently in healthy individuals (Moriyama et al., 2008; Païssé et al., 2016). These findings suggest that alterations in the blood microbiome observed in neurological disorders with gastrointestinal origins may reflect dysbiosis patterns similar to those seen in other systemic diseases (**Figure 1**).

Additionally, Ciocan et al. identified blood dysbiosis in patients with untreated major depressive episodes, marked by reduced Fusobacteria and Candidatus Saccharibacteria, enriched *Janthinobacterium*, and diminished *Neisseria*. Increased Firmicutes and decreased Proteobacteria and Actinobacteria were linked to positive treatment response (Ciocan et al., 2021). Liu et al. reported blood dysbiosis in Parkinson’s disease (PD), noting increased *Myroides, Isoptericola, Microbacterium, Cloacibacterium*, and *Enhydrobacter*, with reduced *Limnobacter* levels (Liu et al., 2021). Pérez-Soriano et al. found that the blood of Multiple System Atrophy (MSA) patients exhibited elevated microbiome levels, with distinct bacterial profiles for each subtype. For instance, cerebellar MSA showed increased *Acinetobacter* and decreased *Blastococcus* and *Bacillus* compared to PD (Pérez-soriano et al., 2020). However, these studies are observational, and more comprehensive cohort studies are needed to uncover new etiologies of neurological disorders associated with the blood microbiota. Such research could also aid in identifying diagnostic biomarkers and promising therapeutic strategies targeting blood microbiome dysbiosis in these conditions.

## Alterations in the blood microbiome in immune-mediated diseases

Autoimmune diseases have a substantial impact on health, quality of life, healthcare usage, and the economy, resulting in increased mortality. Despite their rarity, they collectively affect 1 in 31 Americans and are a leading cause of death in young and middle-aged women (Jacobson et al., 1997; Walsh and Rau, 2000). Unlike diseases sharing common underlying causes, such as cancers or CVD, autoimmune disorders have typically been viewed as distinct entities, and their origins are largely unknown (Cooper and Stroehla, 2003). Global organizations have, therefore, underlined the necessity of population-based epidemiologic studies on autoimmune diseases (Committee, 2005).

Recent studies show that individuals with known or suspected autoimmune diseases have abnormal blood microbiome profiles that differ consistently from those of healthy individuals. Ogunrinde et al. reported that anti-double-stranded DNA antibodies and anti-nuclear factors play a role in systemic lupus erythematosus (SLE). Remarkably, depleted levels of *Paenibacillus* were detected in the blood of SLE patients and their first-degree relatives than those of healthy individuals (Ogunrinde et al., 2019). These findings suggest that genetic factors might be associated with this correlation. In contrast, Luo et al. observed elevated levels of *Planococcus* in patients with SLE. Exposure of peripheral blood mononuclear cells to *Planococcus* triggered the release of significant inflammatory cytokines, potentially contributing to the chronic inflammation characteristic of SLE (Alekseyenko et al., 2021). Jones et al. reported elevated levels of Cytophagia in the blood, indicating dysbiosis in patients with large vessel vasculitis, such as giant cell arteritis and Takayasu’s arteritis, compared to controls. In Takayasu’s arteritis, the presence of *Staphylococcus* in the blood may further exacerbate the condition (Jones et al., 2021). Cheng et al. reported that anti-rheumatic medications used to treat rheumatoid arthritis (RA) may potentially reverse blood dysbiosis by increasing the levels of *Corynebacterium* and *Streptococcus* while decreasing *Shewanella* (Cheng et al., 2023). *Shewanella* may contribute to RA pathogenesis through immune activation via its lipopolysaccharides, induction of pro-inflammatory cytokines, or molecular mimicry triggering autoimmunity, though direct causal evidence remains limited. Blood microbiome dysbiosis in RA may involve an elevated level of Lachnospiraceae, *Halomonas*, and *Shewanella*, while *Corynebacterium*1 and *Streptococcus* may decline (Chang et al., 2021). Lachnospiraceae appeared to continue elevation even after therapy, possibly indicating a compensatory response to blood dysbiosis. The elevation in Lachnospiraceae might have a positive impact on the course of the disease. In contrast, Puri et al. reported reduced levels of Firmicutes and Fusobacteria in the blood of psoriasis patients (Puri et al., 2018), while Han et al. stated that the pathophysiology of RA may involve a higher level of genus *Pelagibacterium* and PARP9 mRNA (Han and Lo, 2021). Despite common etiologies, blood microbiome configurations diverge across autoimmune diseases. In immune-mediated reversible obstructive airway disease and asthma, dysbiosis was characterized by a Bacteroidetes-enriched signature (Buford et al., 2018; Koliarakis et al., 2020). During airway inflammation, the lung microbiome, typically enriched in Bacteroidetes, may translocate into the bloodstream, reshaping the blood microbiome signature in these conditions (Zhang et al., 2019; Zhu et al., 2020). The long-term steroid treatment for airway constriction reduced *Staphylococcus* and *Rothia*, potentially altering the blood mirobiome in asthma (**Figure 1**). Systemic steroids were linked to increased levels of *Prevotella 9, Intestinibacter, Lactobacillus*, and *Blautia* (Buford et al., 2018). Large-scale, multi-cohort studies are needed to validate the contribution of identified taxa to disease progression and to uncover potential therapeutic targets.

## Variability in blood microbiome study designs and their impact

Variability in blood microbiome study designs poses a significant challenge to establishing consistent microbial signatures linked to disease. Understanding these sources of variation is crucial for advancing the field and improving reproducibility.

- Methodological heterogeneity: Variations in blood sampling, DNA extraction, sequencing platforms, and bioinformatics workflows affect microbial detection and quantification (Regueira‐Iglesias et al., 2023), driving inconsistent results.
- Population diversity: Differences in cohort demographics, genetics, disease stages, and treatment history shape blood microbiome profiles (Gilbert et al., 2018), complicating cross-study comparisons.
- Temporal Dynamics: The blood microbiome fluctuates over time due to factors like diet, medication, and disease progression (Halfvarson et al., 2017), causing variability in findings from different sampling points.
- Environmental Influences: Geographic and ecological exposures modulate systemic microbiomes (Gacesa et al., 2022), adding variability across study populations.
- Analytical Focus: Targeted approaches emphasizing specific taxa may miss broader microbial shifts (Hanson et al., 2012), leading to divergent conclusions across studies.

Addressing these variables through standardized methodologies, comprehensive cohort characterization, longitudinal sampling, and unbiased analytical frameworks is essential to enhance reproducibility and accurately define the blood microbiome’s role in disease pathogenesis.

## Vulnerability of low-biomass samples to contaminants

The extent of contaminant DNA and cross-contamination varies depending on the microbial biomass of each sample. Microbial biomass can be estimated by comparing microbial DNA quantities, such as 16S rRNA gene copies measured by qPCR, in samples versus DNA extraction blank controls (Lauder et al., 2016). High-biomass samples, like feces and soil, contain significantly more microbial DNA than blanks, whereas low-biomass samples, including blood, placenta, air, and built environments, often have DNA levels comparable to blanks. In low-biomass samples, the limited microbial DNA makes them highly susceptible to contamination from exogenous DNA or cross-contamination during processing, especially when handled alongside high-biomass samples, leading to false or misleading microbial profiles (Salter et al., 2014; Glassing et al., 2016; Lauder et al., 2016).

Many researchers question the blood microbiome’s existence because low-biomass samples are easily contaminated at multiple steps during processing (Chrisman et al., 2022). Several studies suggest that the blood microbiome in healthy individuals likely traces of microbial DNA from external sources or remnants of non-viable bacteria (Glassing et al., 2016; Martel et al., 2017). For similar reasons, claims of resident microbiomes in traditionally sterile, low-biomass niches, such as the prenatal womb, central nervous system, and tumor microenvironments, have been increasingly challenged and remain contentious (Kennedy et al., 2023). NGS is highly sensitive to trace microbial DNA contaminants originating from extraction kits, storage vessels, reagents, and even sequencing platforms. Additionally, skin-derived bacteria introduced during venepuncture and handling errors by clinical staff can further confound results. Most blood microbiome studies lack rigorous reporting on decontamination strategies, and even when addressed, efforts are often insufficient; comprehensive negative controls across all workflow stages are likely essential. Integrating bioinformatic and statistical tools into decontamination workflows has been proposed to enhance the accuracy of low-biomass microbiome profiling and reduce false-positive microbial signals (Davis et al., 2018). Therefore, establishing robust and contamination-resilient methodologies is fundamental for advancing microbiome research in low-biomass environments like the bloodstream.

## Pitfalls in low-biomass metagenomics for blood microbiome research

Metagenomics enables culture-independent profiling of the blood microbiome (“hemobiome”) (Govender, 2024), but its application in low-biomass environments like blood is technically challenging. Microbial DNA comprises less than 0.1% of total nucleic acids and is easily masked by host DNA and environmental contaminants (Païssé et al., 2016; Selway et al., 2020). Artefacts introduced by reagents, lab surfaces, PCR biases, index hopping, and sequencing limitations can inflate false positives and compromise reproducibility (Païssé et al., 2016). Païssé et al. (2016) further reveal a highly diverse and quantitatively significant blood microbiome in healthy donors, varying across individuals and blood fractions. While molecular tools like qPCR and sequencing may help detect transfusion-transmitted bacterial infections, especially in immunocompromised recipients, their high sensitivity risks misclassifying clinically irrelevant DNA (Païssé et al., 2016). These findings underscore the need for cautious interpretation and standardized thresholds in low-biomass metagenomics to avoid false positives and guide safe, evidence-based blood screening practices. Furthermore, recent AI-based methods, particularly deep learning and ensemble classifiers (e.g., Random Forests, Gradient Boosting), offer a promising framework to overcome these limitations (Wani et al., 2022). These models can learn contamination patterns, filter noise, and extract biologically relevant features from noisy, sparse datasets. They also enable integration of heterogeneous inputs, such as metagenomic profiles, clinical metadata, and host response markers, to improve diagnostic precision. Some models have been trained to differentiate viable pathogens from remnant DNA fragments, further refining signal interpretation. As demonstrated by Wani et al. (2022), these approaches enhance the detection of clinically relevant taxa such as *Staphylococcus* spp. and position the hemobiome as a viable substrate for liquid biopsy and microbiome-guided precision diagnostics. For clinical application of metagenomic sequencing, rigorous interpretive thresholds must be established using metrics such as read counts, relative abundance, read quality scores, depth of coverage, and results from external and internal controls processed alongside clinical samples (Vijayvargiya et al., 2019). Given the inherent background noise and the difficulty of fully eliminating non-target microbial reads, metagenomic sequencing may be limited in reliably detecting low-abundance pathogens, particularly in low-biomass contexts like blood.

Unlike well-characterized niches such as the gut or skin, the blood microbiome remains poorly defined and highly contentious (Velmurugan et al., 2020; Chrisman et al., 2022). The technical pitfalls outlined above, especially contamination, low microbial load, and overreliance on DNA-based detection, contribute to inconsistent findings across studies. Discrepancies in reported microbial profiles raise fundamental questions about whether the signals reflect viable communities, transient translocation, or residual cell-free DNA (Glassing et al., 2016; Martel et al., 2017). Although rigorous controls and standardized workflows improve detection fidelity, they cannot fully eliminate the risk of artefactual signals (Jervis-Bardy et al., 2015; Davis et al., 2018). These challenges mirror debates in other low-biomass sites like the placenta, brain, and tumors (Kennedy et al., 2023), where microbial detection remains controversial. Crucially, next-generation sequencing lacks the resolution to confirm microbial viability or origin, limiting its interpretive value (Panaiotov et al., 2021). Emerging RNA-based methods, such as FISH, PETRI-seq, MATQ-seq, and BacDrop, enable single-cell resolution and functional assessment of microbial activity (Batani et al., 2019; Kuchina et al., 2021; Cheng et al., 2023; Homberger et al., 2023; Ma et al., 2023). These tools may help distinguish true microbial residents from technical artefacts or biological noise. Ultimately, realizing the potential of blood metagenomics will require methodological rigor, functional validation, and integration of multi-omic data to overcome the pitfalls inherent to low-biomass microbiome research.

## Contamination in microbiome research: sources, dynamics, and impact on data integrity

Microbiome studies face two main contamination challenges: contaminant DNA and cross-contamination. Contaminant DNA can originate from various sources despite rigorous sample collection and processing protocols, including the sampling environment, laboratory settings (Llamas et al., 2017), personnel, plasticware (Motley et al., 2014), nucleic acid extraction kits (Glassing et al., 2016; Weyrich et al., 2017), and reagents such as PCR mastermixes (Shen et al., 2006; Eisenhofer et al., 2019). Additionally, contamination may arise from other samples or sequencing runs (Seitz et al., 2015; Ballenghien et al., 2017). More than 60 common contaminant taxa have been identified repeatedly in DNA extraction blanks and no-template controls across multiple studies. For instance, Salter et al. demonstrated that several contaminant taxa were consistently detected across different labs, extraction methods, and studies (Salter et al., 2014). These pervasive contaminants likely originate from sources such as kit and reagent manufacturing, human commensals on laboratory staff, and environmental exposure. However, contaminant profiles vary depending on extraction kits, laboratory environments (Glassing et al., 2016; Weyrich et al., 2017), and even fluctuate over time within the same lab (Weyrich et al., 2019).

Cross-contamination poses a further obstacle during microbiome sample processing and involves the unintended transfer of sample DNA, barcodes, or amplicons between adjacent wells or tubes, leading to batch effects (Nguyen et al., 2015). This can occur at various stages, including sample handling and tube or plate loading (Tamariz et al., 2006), as well as through aerosolization during pipetting or plate cover removal (Mainelis, 2020). Barcode contamination can arise when incorrect barcodes “jump” into neighboring samples, a process termed ‘tag switching’ (Carlsen et al., 2012). Additionally, cross-contamination may occur on sequencing platforms due to barcode sequencing errors, residual amplicons from previous runs, or ‘index hopping,’ where indexing reads are incorrectly assigned to sequencing reads (Larsson et al., 2018). Both contaminant DNA and cross-contamination are dynamic challenges that require continuous and careful monitoring throughout microbiome research workflows.

## Blood microbiome research: unresolved challenges, controversies, and translational barriers

The study of the blood microbiome across various disease states has revealed microbial signatures linked to diagnosis, disease severity, and prognosis. Despite inconsistent findings, efforts to define blood dysbiosis are advancing, addressing key questions: “Who is there?” and “What do they do?” Current data suggest a predominance of bacteria, with a clear taxonomic structure in the blood microbiome, primarily composed of Proteobacteria, Bacteroidetes, Firmicutes, and Actinobacteria, which have been observed in blood across different health conditions (Amar et al., 2019; Shah et al., 2019; Scarsella et al., 2020; Khan et al., 2022b, 2022). However, the specific functions of distinct blood microbiome profiles and their roles in disease mechanisms remain largely unexplored. Given its potential public health impact, the blood microbiome warrants increased attention, as it has implications for both human and animal health. This perspective mirrors Tolstoy’s “Anna Karenina principle, “happy families are all alike; every unhappy family is unhappy in its own way.” Similarly, while a common foundation for health maintenance may exist within the blood microbiome, variations in microbial profiles may uniquely contribute to different pathological conditions, underscoring the need for targeted interventions and in-depth research (Zaneveld et al., 2017). Our understanding of the blood microbiome’s role in health and disease is still nascent, with significant gaps remaining in defining a “core blood microbiome,” understanding its health benefits, and elucidating its specific functions.

Despite growing interest in the blood microbiome, several studies report null or contradictory findings regarding microbial presence and associations with disease. Variations in sampling methods, contamination risks, and analytical techniques contribute to inconsistent results. Some studies fail to detect microbial signatures in blood, while others report conflicting microbial taxa linked to similar conditions. These discrepancies highlight the need for standardized protocols and rigorous controls to validate findings reliably. Future research on the blood microbiome should explore new areas while deepening our understanding of its mechanisms. Clarifying its role in disease progression, systemic inflammation, and comorbidities could revolutionize diagnostics and therapies. While current studies focus on microbial associations with systemic diseases, few have investigated interventions targeting the blood microbiome. Future studies should prioritize controlled trials, such as antimicrobial treatments, probiotics, or immune-modulating strategies, to assess causality and therapeutic potential. A better understanding of microbial-immune interactions is essential, especially in systemic inflammation and disease. Future research should focus on the molecular interactions between blood microbes and immune responses to develop targeted therapies. Personalized blood microbiome-based treatments hold promise, as tailored interventions may be more effective than generic ones. Identifying harmful microbial signatures linked to diseases could lead to targeted therapies, including bacteriophage treatment, which could reduce reliance on broad-spectrum antibiotics and combat antibiotic resistance. These precision strategies have the potential to transform disease prevention and treatment.

These insights pave the way for blood-based diagnostics and targeted microbiome-metabolome interventions. Biomarkers derived from circulating microbial taxa and metabolites hold promise for early disease detection. Therapeutically, metabolite supplementation (e.g., SCFAs, bile acids), antibiotics, probiotics, or vaccines (e.g., BCG) could modulate host–microbe interactions and inflammation. To unlock the full therapeutic potential of the blood microbiome, we must explore its “dark matter,” including the virome and uncultivated microbial taxa with functional significance that are not yet fully understood (Rodríguez del Río et al., 2024). However, challenges include distinguishing viable microbes from DNA fragments, understanding causal mechanisms, ensuring brain access, and improving omics integration. Longitudinal, multi-omic studies are essential to clarify whether these blood microbiome and metabolome changes are drivers or just bystanders in systemic disease.

## Conclusion

The blood microbiome is increasingly recognized as a key player in the pathogenesis of diverse systemic diseases, including infectious, neurological, and immune-mediated conditions, through its influence on systemic inflammation, immune modulation, and metabolic disruption. Defining clear clinical outcomes and mechanistic parameters is essential to advance both research and therapeutic applications. However, current progress is hindered by methodological pitfalls such as inadequate contamination control, inconsistent sequencing protocols, and a lack of viability assessment. To overcome these barriers, rigorously designed, longitudinal multi-omic studies are needed to clarify causal relationships and enhance biomarker specificity. As we move beyond traditional approaches like probiotics and bacteriophage therapy, future translational success will rely on innovative, mechanistically guided microbiome-based interventions tailored to disease-specific blood microbial signatures, ultimately improving risk prediction, treatment, and patient outcomes.

## References

[B1] Alekseyenko A. V. Ogunrinde E. Li M. Tsao B. P. Kamen D. L. Oates J. C.. (2021). Rigorous plasma microbiome analysis method enables disease association discovery in clinic. Front. Microbiol. 11. doi: 10.3389/fmicb.2020.613268, PMID: 33488555 PMC7820181

[B2] Amar J. Lelouvier B. Servant F. Lluch J. Burcelin R. Bongard V.. (2019). Blood microbiota modification after myocardial infarction depends upon low-density lipoprotein cholesterol levels. J. Am. Heart Assoc. 8, e011797. doi: 10.1161/JAHA.118.011797, PMID: 31566105 PMC6806051

[B3] Ancona A. Petito C. Iavarone I. Petito V. Galasso L. Leonetti A.. (2021). The gut–brain axis in irritable bowel syndrome and inflammatory bowel disease. Dig. Liver Dis. 53, 298–305. doi: 10.1016/j.dld.2020.11.026, PMID: 33303315

[B4] Ballenghien M. Faivre N. Galtier N. (2017). Patterns of cross-contamination in a multispecies population genomic project: detection, quantification, impact, and solutions. BMC Biol. 15, 1–16. doi: 10.1186/s12915-017-0366-6, PMID: 28356154 PMC5370491

[B5] Batani G. Bayer K. Böge J. Hentschel U. Thomas T. (2019). Fluorescence in *situ* hybridization (FISH) and cell sorting of living bacteria. Sci. Rep. 9, 18618. doi: 10.1038/s41598-019-55049-2, PMID: 31819112 PMC6901588

[B6] Buford T. W. Carter C. S. VanDerPol W. J. Chen D. Lefkowitz E. J. Eipers P.. (2018). Composition and richness of the serum microbiome differ by age and link to systemic inflammation. Geroscience 40, 257–268. doi: 10.1007/s11357-018-0026-y, PMID: 29869736 PMC6060185

[B7] Carlsen T. Aas A. B. Lindner D. Vrålstad T. Schumacher T. Kauserud H. (2012). Don’t make a mista (g) ke: is tag switching an overlooked source of error in amplicon pyrosequencing studies? Fungal Ecol. 5, 747–749. doi: 10.1016/j.funeco.2012.06.003

[B8] Castillo D. J. Rifkin R. F. Cowan D. A. Potgieter M. (2019). The healthy human blood microbiome: Fact or fiction? Front. Cell. Infect. Microbiol. 9. doi: 10.3389/fcimb.2019.00148, PMID: 31139578 PMC6519389

[B9] Chang C.-J. Zhang J. Tsai Y.-L. Chen C.-B. Lu C.-W. Huo Y. P.. (2021). Compositional features of distinct microbiota base on serum extracellular vesicle metagenomics analysis in moderate to severe psoriasis patients. Cells 10, 2349. doi: 10.3390/cells10092349, PMID: 34571998 PMC8467001

[B10] Chen Y. Zhou J. Wang L. (2021). Role and mechanism of gut microbiota in human disease. Front. Cell. Infect. Microbiol. 11, 625913. doi: 10.3389/fcimb.2021.625913, PMID: 33816335 PMC8010197

[B11] Cheng H. S. Tan S. P. Meng D. Wong K. Ling W. Koo Y.. (2023). The blood microbiome and health : current evidence, controversies, and challenges. Intl. J. Mol. Sci. 24 (6), 5633. doi: 10.3390/ijms24065633, PMID: 36982702 PMC10059777

[B12] Chrisman B. He C. Jung J.-Y. Stockham N. Paskov K. Washington P.. (2022). The human “contaminome”: bacterial, viral, and computational contamination in whole genome sequences from 1000 families. Sci. Rep. 12, 9863. doi: 10.1038/s41598-022-13269-z, PMID: 35701436 PMC9198055

[B13] Ciocan D. Cassard A. Becquemont L. Verstuyft C. Voican C. S. Asmar K.. (2021). Blood microbiota and metabolomic signature of major depression before and after antidepressant treatment : a prospective case – control study. J. Psychiatry Neurosci. 46, 358–368. doi: 10.1503/jpn.200159, PMID: 34008933 PMC8327971

[B14] Committee A. D. C. (2005). Progress in autoimmune diseases research: Report to Congress. US Dep. Heal. Hum. Serv.

[B15] Cooper G. S. Stroehla B. C. (2003). The epidemiology of autoimmune diseases. Autoimmun. Rev. 2, 119–125. doi: 10.1016/S1568-9972(03)00006-5, PMID: 12848952

[B16] Damgaard C. Magnussen K. Enevold C. Nilsson M. Tolker-Nielsen T. Holmstrup P.. (2015). Viable bacteria associated with red blood cells and plasma in freshly drawn blood donations. PloS One 10, e0120826. doi: 10.1371/journal.pone.0120826, PMID: 25751254 PMC4353618

[B17] Davis N. M. Proctor D. M. Holmes S. P. Relman D. A. Callahan B. J. (2018). Simple statistical identification and removal of contaminant sequences in marker-gene and metagenomics data. Microbiome 6, 1–14. doi: 10.1186/S40168-018-0605-2, PMID: 30558668 PMC6298009

[B18] Dereschuk K. Apostol L. Ranjan I. Chakladar J. Li W. T. Rajasekaran M.. (2021). Identification of lung and blood microbiota implicated in COVID-19 prognosis. Cells 10, 1452. doi: 10.3390/cells10061452, PMID: 34200572 PMC8226556

[B19] Dinakaran V. Rathinavel A. Pushpanathan M. Sivakumar R. Gunasekaran P. Rajendhran J. (2014). Elevated levels of circulating DNA in cardiovascular disease patients: Metagenomic profiling of microbiome in the circulation. PloS One 9, e105221. doi: 10.1371/journal.pone.0105221, PMID: 25133738 PMC4136842

[B20] Eisenhofer R. Minich J. J. Marotz C. Cooper A. Knight R. Weyrich L. S. (2019). Contamination in low microbial biomass microbiome studies: issues and recommendations. Trends Microbiol. 27, 105–117. doi: 10.1016/j.tim.2018.11.003, PMID: 30497919

[B21] Finland S. (2021). Official statistics of Finland (OSF): Causes of death. (Official Statistics of Finland (OSF))

[B22] Funkhouser L. J. Bordenstein S. R. (2013). Mom knows best: the universality of maternal microbial transmission. PloS Biol. 11, e1001631. doi: 10.1371/journal.pbio.1001631, PMID: 23976878 PMC3747981

[B23] Gacesa R. Kurilshikov A. Vich Vila A. Sinha T. Klaassen M. A. Y. Bolte L. A.. (2022). Environmental factors shaping the gut microbiome in a Dutch population. Nature 604, 732–739. doi: 10.1038/s41586-022-04567-7, PMID: 35418674

[B24] Gedgaudas R. Bajaj J. S. Skieceviciene J. Varkalaite G. Jurkeviciute G. Gelman S.. (2022). Circulating microbiome in patients with portal hypertension. Gut Microbes 14, 2029674. doi: 10.1080/19490976.2022.2029674/SUPPL_FILE/KGMI_A_2029674_SM1876.ZIP, PMID: 35130114 PMC8824227

[B25] Gilbert J. A. Blaser M. J. Caporaso J. G. Jansson J. K. Lynch S. V. Knight R. (2018). Current understanding of the human microbiome. Nat. Med. 24, 392–400. doi: 10.1038/nm.4517, PMID: 29634682 PMC7043356

[B26] Glassing A. Dowd S. E. Galandiuk S. Davis B. Chiodini R. J. (2016). Inherent bacterial DNA contamination of extraction and sequencing reagents may affect interpretation of microbiota in low bacterial biomass samples. Gut Pathog. 8, 1–12. doi: 10.1186/S13099-016-0103-7, PMID: 27239228 PMC4882852

[B27] Gosiewski T. Huminska K. (2017). Comprehensive detection and identification of bacterial DNA in the blood of patients with sepsis and healthy volunteers using next-generation sequencing method - the observation of DNAemia. Eur. J. Clin. Microbiol. Infect. Dis. 36, 329–336. doi: 10.1007/s10096-016-2805-7, PMID: 27771780 PMC5253159

[B28] Govender K. N. (2024). Metagenomic sequencing directly from blood culture as a tool for clinical diagnosis [Doctoral dissertation]. University of Oxford.

[B29] Grochowska M. Laskus T. Radkowski M. (2019). Gut microbiota in neurological disorders. Arch. Immunol. Ther. Exp. (Warsz) 67, 375–383. doi: 10.1007/s00005-019-00561-6, PMID: 31578596 PMC6805802

[B30] Guo X. Wang Z. Qu M. Guo Y. Yu M. Hong W.. (2023). Abnormal blood microbiota profiles are associated with inflammation and immune restoration in HIV/AIDS individuals. Msystems 8 (5), e00467–23. doi: 10.1128/msystems.00467-23, PMID: 37698407 PMC10654078

[B31] Halfvarson J. Brislawn C. J. Lamendella R. Vázquez-Baeza Y. Walters W. A. Bramer L. M.. (2017). Dynamics of the human gut microbiome in inflammatory bowel disease. Nat. Microbiol. 2, 1–7. doi: 10.1038/nmicrobiol.2017.4, PMID: 28191884 PMC5319707

[B32] Hammad D. B. M. Hider S. L. Liyanapathirana V. C. Tonge D. P. (2020). Molecular characterization of circulating microbiome signatures in rheumatoid arthritis. Front. Cell. Infect. Microbiol. 9. doi: 10.3389/fcimb.2019.00440, PMID: 32039040 PMC6987042

[B33] Han D. S. C. Lo Y. M. D. (2021). The nexus of cfDNA and nuclease biology. Trends Genet. 37, 758–770. doi: 10.1016/j.tig.2021.04.005, PMID: 34006390

[B34] Hanson C. A. Fuhrman J. A. Horner-Devine M. C. Martiny J. B. H. (2012). Beyond biogeographic patterns: processes shaping the microbial landscape. Nat. Rev. Microbiol. 10, 497–506. doi: 10.1038/nrmicro2795, PMID: 22580365

[B35] Heintz-Buschart A. Wilmes P. (2018). Human gut microbiome: function matters. Trends Microbiol. 26, 563–574. doi: 10.1016/j.tim.2017.11.002, PMID: 29173869

[B36] Hodzhev Y. (2023). Analysis of blood microbiome dysbiosis in pulmonary sarcoidosis by decision tree model. Biotechnol. Biotechnol. Equip. 37, 2283133. doi: 10.1080/13102818.2023.2283133

[B37] Hodzhev Y. Tsafarova B. Tolchkov V. Youroukova V. Ivanova S. Kostadinov D.. (2023). Visualization of the individual blood microbiome to study the etiology of sarcoidosis. Comput. Struct. Biotechnol. J. 22, 50–57. doi: 10.1016/j.csbj.2023.10.027, PMID: 37928975 PMC10624578

[B38] Homberger C. Hayward R. J. Barquist L. Vogel J. (2023). Improved bacterial single-cell RNA-seq through automated MATQ-seq and Cas9-based removal of rRNA reads. MBio 14, e03557–e03522. doi: 10.1128/MBIO.03557-22, PMID: 36880749 PMC10127585

[B39] Hyun H. Lee M. S. Park I. Ko H. S. Yun S. Jang D.. (2021). Analysis of porcine model of fecal- induced peritonitis reveals the tropism of blood microbiome. Front. Cell. Infect. Microbiol. 11, 1–14. doi: 10.3389/fcimb.2021.676650, PMID: 34527598 PMC8435847

[B40] Iannone L. F. Preda A. Blottière H. M. Clarke G. Albani D. Belcastro V.. (2019). Microbiota-gut brain axis involvement in neuropsychiatric disorders. Expert Rev. Neurother. 19, 1037–1050. doi: 10.1080/14737175.2019.1638763, PMID: 31260640

[B41] Jacobson D. L. Gange S. J. Rose N. R. Graham N. M. H. (1997). Epidemiology and estimated population burden of selected autoimmune diseases in the United States. Clin. Immunol. Immunopathol. 84, 223–243. doi: 10.1006/clin.1997.4412, PMID: 9281381

[B42] Jagare L. Rozenberga M. Silamikelis I. Ansone L. Elbere I. Briviba M.. (2023). Metatranscriptome analysis of blood in healthy individuals and irritable bowel syndrome patients. J. Med. Microbiol. 72 (6), 1–12. doi: 10.1099/jmm.0.001719, PMID: 37335601

[B43] James W. A. Ogunrinde E. Wan Z. Kamen D. L. Oates J. Gilkeson G. S.. (2022). A distinct plasma microbiome but not gut microbiome in patients with systemic lupus erythematosus compared to healthy individuals. J. Rheumatol. 49, 592–597. doi: 10.3899/jrheum.210952, PMID: 35169056 PMC9364828

[B44] Jervis-Bardy J. Leong L. E. X. X. Marri S. Smith R. J. Choo J. M. Smith-Vaughan H. C.. (2015). Deriving accurate microbiota profiles from human samples with low bacterial content through post-sequencing processing of Illumina MiSeq data. Microbiome 3, 1–11. doi: 10.1186/S40168-015-0083-8, PMID: 25969736 PMC4428251

[B45] Jiang W. Lederman M. M. Hunt P. Sieg S. F. Haley K. Rodriguez B.. (2009). Plasma levels of bacterial DNA correlate with immune activation and the magnitude of immune restoration in persons with antiretroviral-treated HIV infection. J. Infect. Dis. 199, 1177–1185. doi: 10.1086/597476, PMID: 19265479 PMC2728622

[B46] Jones E. Stentz R. Telatin A. Savva G. M. Booth C. Baker D.. (2021). The origin of plasma-derived bacterial extracellular vesicles in healthy individuals and patients with inflammatory bowel disease: A pilot study. Genes 12(10), 1636., PMID: 34681030 10.3390/genes12101636 PMC8535827

[B47] Kajihara M. Koido S. Kanai T. Ito Z. Matsumoto Y. Takakura K.. (2019). Characterisation of blood microbiota in patients with liver cirrhosis. Eur. J. Gastroenterol. Hepatol. 31, 1577–1583. doi: 10.1097/MEG.0000000000001494, PMID: 31441799 PMC6844652

[B48] Kennedy K. M. de Goffau M. C. Perez-Muñoz M. E. Arrieta M.-C. Bäckhed F. Bork P.. (2023). Questioning the fetal microbiome illustrates pitfalls of low-biomass microbial studies. Nature 613, 639–649. doi: 10.1038/s41586-022-05546-8, PMID: 36697862 PMC11333990

[B49] Khan I. I. I. I. I. Khan I. I. I. I. I. Jianye Z. Xiaohua Z. Khan M. Gul M.. (2022a). Exploring blood microbial communities and their influence on human cardiovascular disease. J. Clin. Lab. Anal. 36, 1–11. doi: 10.1002/jcla.24354, PMID: 35293034 PMC8993628

[B50] Khan I. Khan I. Kakakhel M. A. Xiaowei Z. Ting M. Ali I.. (2022). Comparison of microbial populations in the blood of patients with myocardial infarction and healthy individuals. Front. Microbiol. 13. doi: 10.3389/fmicb.2022.845038, PMID: 35694288 PMC9176212

[B51] Khan I. Khan I. Usman M. Jianye Z. Wei Z. X. Ping X.. (2022b). Analysis of the blood bacterial composition of patients with acute coronary syndrome and chronic coronary syndrome. Front. Cell. Infect. Microbiol. 12. doi: 10.3389/fcimb.2022.943808, PMID: 36268223 PMC9577097

[B52] Koliarakis I. Athanasakis E. Sgantzos M. Mariolis-Sapsakos T. Xynos E. Chrysos E.. (2020). Intestinal microbiota in colorectal cancer surgery. Cancers (Basel) 12, 3011. doi: 10.3390/cancers12103011, PMID: 33081401 PMC7602998

[B53] KuChina A. Brettner L. M. Paleologu L. Roco C. M. Rosenberg A. B. Carignano A.. (2021). Microbial single-cell RNA sequencing by split-pool barcoding. Sci. (80-.) 371, eaba5257. doi: 10.1126/SCIENCE.ABA5257, PMID: 33335020 PMC8269303

[B54] Larsson A. J. M. Stanley G. Sinha R. Weissman I. L. Sandberg R. (2018). Computational correction of index switching in multiplexed sequencing libraries. Nat. Methods 15, 305–307. doi: 10.1038/nmeth.4666, PMID: 29702636

[B55] Lauder A. P. Roche A. M. Sherrill-Mix S. Bailey A. Laughlin A. L. Bittinger K.. (2016). Comparison of placenta samples with contamination controls does not provide evidence for a distinct placenta microbiota. Microbiome 4, 1–11. doi: 10.1186/s40168-016-0172-3, PMID: 27338728 PMC4917942

[B56] Lee J.-H. H. Choi J.-P. P. Yang J. Won H.-K. K. Park C. S. Song W.-J. J.. (2020). Metagenome analysis using serum extracellular vesicles identified distinct microbiota in asthmatics. Sci. Rep. 10, 1–9. doi: 10.1038/s41598-020-72242-w, PMID: 32934287 PMC7492258

[B57] Lelouvier B. Servant F. Païssé S. Brunet A. C. Benyahya S. Serino M.. (2016). Changes in blood microbiota profiles associated with liver fibrosis in obese patients: A pilot analysis. Hepatology 64, 2015–2027. doi: 10.1002/hep.28829, PMID: 27639192

[B58] Li Q. Wang C. Tang C. Zhao X. He Q. Li J. (2018). Identification and characterization of blood and neutrophil-associated microbiomes in patients with severe acute pancreatitis using next-generation sequencing. Front. Cell. Infect. Microbiol. 8. doi: 10.3389/fcimb.2018.00005, PMID: 29423379 PMC5790034

[B59] Liang G. Bushman F. D. Liang G. (2021). The human virome : assembly, composition and host interactions. Nat. Rev. Microbiol. 19, 514–527. doi: 10.1038/s41579-021-00536-5, PMID: 33785903 PMC8008777

[B60] Libertucci J. Young V. B. (2019). The role of the microbiota in infectious diseases. Nat. Microbiol. 4, 35–45. doi: 10.1038/s41564-018-0278-4, PMID: 30546094

[B61] Liu X. Tang S. Zhong H. Tong X. Jie Z. Ding Q.. (2021). A genome-wide association study for gut metagenome in Chinese adults illuminates complex diseases. Cell Discov. 7, 9. doi: 10.1038/s41421-020-00239-w, PMID: 33563976 PMC7873036

[B62] Llamas B. Valverde G. Fehren-Schmitz L. Weyrich L. S. Cooper A. Haak W. (2017). From the field to the laboratory: Controlling DNA contamination in human ancient DNA research in the high-throughput sequencing era. STAR Sci. Technol. Archaeol. Res. 3, 1–14. doi: 10.1080/20548923.2016.1258824

[B63] Olde Loohuis L. M Mangul S. Ori A. P. S. Jospin G. Koslicki D. Yang H. T.. (2018). Transcriptome analysis in whole blood reveals increased microbial diversity in schizophrenia. Transl. Psychiatry. 8, 1–9. doi: 10.1038/s41398-018-0107-9, PMID: 29743478 PMC5943399

[B64] Lucchinetti E. Lou P.-H. Lemal P. Bestmann L. Hersberger M. Rogler G.. (2022). Gut microbiome and circulating bacterial DNA (“blood microbiome”) in a mouse model of total parenteral nutrition: Evidence of two distinct separate microbiotic compartments. Clin. Nutr. ESPEN 49, 278–288. doi: 10.1016/j.clnesp.2022.03.038, PMID: 35623826

[B65] Luo Z. Li M. Wu Y. Meng Z. Martin L. Zhang L.. (2019). Systemic translocation of Staphylococcus drives autoantibody production in HIV disease. Microbiome 7, 1–16. doi: 10.1186/s40168-019-0646-1, PMID: 30764863 PMC6376754

[B66] Ma P. Amemiya H. M. He L. L. Gandhi S. J. Nicol R. Bhattacharyya R. P.. (2023). Bacterial droplet-based single-cell RNA-seq reveals antibiotic-associated heterogeneous cellular states. Cell 186, 877–891. doi: 10.1016/j.cell.2023.01.002, PMID: 36708705 PMC10014032

[B67] Mainelis G. (2020). Bioaerosol sampling: Classical approaches, advances, and perspectives. Aerosol Sci. Technol. 54, 496–519. doi: 10.1080/02786826.2019.1671950, PMID: 35923417 PMC9344602

[B68] Markova N. (2020). Eubiotic vs. Dysbiotic Human Blood Microbiota: the Phenomenon of Cell Wall Deficiency and Disease-trigger Potential of Bacterial and Fungal L-forms. Discovery Med. 29 (156), 17–26., PMID: 32598861

[B69] Martel J. Wu C.-Y. C. Huang P. P.-R. Cheng W. W.-Y. Young J. D. Reports J. Y.-S.. (2017). Pleomorphic bacteria-like structures in human blood represent non-living membrane vesicles and protein particles. Sci. Rep. 7, 10650. doi: 10.1038/s41598-017-10479-8, PMID: 28878382 PMC5587737

[B70] Mayer E. A. Nance K. Chen S. (2022). The gut–brain axis. Annu. Rev. Med. 73, 439–453. doi: 10.1146/annurev-med-042320-014032, PMID: 34669431

[B71] Mo X. Dong C. He P. Wu L. Lu X. (2020). Alteration of circulating microbiome and its associated regulation role in rheumatoid arthritis: Evidence from integration of multiomics data. Clinical and Translational Medicine 10 (7), e229. doi: 10.1002/ctm2.229, PMID: 33252855 PMC7668190

[B72] Mohamed W. Mohamed A. Ali A. O. Mahmoud H. Y. A. H. Omar M. A. Chatanga E.. (2021). Exploring prokaryotic and eukaryotic microbiomes helps in detecting tick-borne infectious agents in the blood of camels. Pathogens 10 (3), 351. doi: 10.3390/pathogens10030351, PMID: 33809738 PMC8002256

[B73] Morais L. H. Schreiber H. L. IV Mazmanian S. K. (2021). The gut microbiota–brain axis in behaviour and brain disorders. Nat. Rev. Microbiol. 19, 241–255. doi: 10.1038/s41579-020-00460-0, PMID: 33093662

[B74] Moriyama K. Ando C. Tashiro K. Kuhara S. Okamura S. Nakano S.. (2008). Polymerase chain reaction detection of bacterial 16S rRNA gene in human blood. Microbiol. Immunol. 52, 375–382. doi: 10.1111/j.1348-0421.2008.00048.x, PMID: 18667036

[B75] Motley S. T. Picuri J. M. Crowder C. D. Minich J. J. Hofstadler S. A. Eshoo M. W. (2014). Improved multiple displacement amplification (iMDA) and ultraclean reagents. BMC Genomics 15, 1–10. doi: 10.1186/1471-2164-15-443, PMID: 24906487 PMC4061449

[B76] Nguyen N. H. Smith D. Peay K. Kennedy P. (2015). Parsing ecological signal from noise in next generation amplicon sequencing. New Phytol. 205, 1389–1393. doi: 10.1111/nph.12923, PMID: 24985885

[B77] Nikkari S. McLaughlin I. J. Bi W. Dodge D. E. Relman D. A. (2001). Does blood of healthy subjects contain bacterial ribosomal DNA? J. Clin. Microbiol. 39, 1956–1959. doi: 10.1128/JCM.39.5.1956-1959.2001, PMID: 11326021 PMC88056

[B78] Ogunrinde E. Zhou Z. Luo Z. Alekseyenko A. Li Q. Macedo D.. (2019). A link between plasma microbial translocation, microbiome, and autoantibody development in first- ­ Degree relatives of systemic lupus erythematosus patients. Arthritis Rheumatol. 71, 1858–1868. doi: 10.1002/art.40935, PMID: 31106972 PMC6817371

[B79] Païssé S. Valle C. Servant F. Courtney M. Burcelin R. Amar J.. (2016). Comprehensive description of blood microbiome from healthy donors assessed by 16S targeted metagenomic sequencing. Transfusion 56, 1138–1147. doi: 10.1111/trf.13477, PMID: 26865079

[B80] Panaiotov S. Filevski G. Equestre M. Nikolova E. Kalfin R. Superiore I.. (2018). Cultural isolation and characteristics of the blood microbiome of healthy individuals. Adv. Microbiol. 8, 406–421. doi: 10.4236/aim.2018.85027

[B81] Panaiotov S. Hodzhev Y. Tsafarova B. Tolchkov V. Kalfin R. (2021). Culturable and non-culturable blood microbiota of healthy individuals. Microorganisms 9, 1464. doi: 10.3390/microorganisms9071464, PMID: 34361900 PMC8304615

[B82] Pérez-soriano A. Segura M. A. Botta-orfila T. (2020). Transcriptomic differences in MSA clinical variants. Sci. Rep. 10 (1), 10310. 1–9. doi: 10.1038/s41598-020-66221-4, PMID: 32587362 PMC7316739

[B83] Poore G. D. Kopylova E. Zhu Q. Carpenter C. Fraraccio S. Wandro S.. (2020). Microbiome analyses of blood and tissues suggest cancer diagnostic approach. Nature 579, 567–574. doi: 10.1038/s41586-020-2095-1, PMID: 32214244 PMC7500457

[B84] Puri P. Liangpunsakul S. Christensen J. E. Shah V. H. Kamath P. S. Gores G. J.. (2018). The circulating microbiome signature and inferred functional metagenomics in alcoholic hepatitis. Hepatology 67, 1284–1302. doi: 10.1002/hep.29623, PMID: 29083504 PMC5867221

[B85] Qian Y. Yang X. Xu S. Wu C. Qin N. Chen S.-D.. (2018). Detection of microbial 16S rRNA gene in the blood of patients with Parkinson’s disease. Front. Aging Neurosci. 10. doi: 10.3389/fnagi.2018.00156, PMID: 29881345 PMC5976788

[B86] Qiu J. Zhou H. Jing Y. Dong C. (2019). Association between blood microbiome and type 2 diabetes mellitus : A nested case - control study. J. Clin. Lab. Anal. 33 (4), e22842. 1–7. doi: 10.1002/jcla.22842, PMID: 30714640 PMC6528574

[B87] Regueira-Iglesias A. Balsa-Castro C. Blanco-Pintos T. Tomás I. (2023). Critical review of 16S rRNA gene sequencing workflow in microbiome studies: From primer selection to advanced data analysis. Mol. Oral. Microbiol. 38, 347–399. doi: 10.1111/omi.12434, PMID: 37804481

[B88] Ring H. C. C. Thorsen J. Saunte D. M. M. Lilje B. Bay L. Theut Riis P.. (2018). Moderate to severe hidradenitis suppurativa patients do not have an altered bacterial composition in peripheral blood compared to healthy controls. J. Eur. Acad. Dermatol. Venereol. 32, 125–128. doi: 10.1111/jdv.14538, PMID: 28833590

[B89] Rodríguez del Río Á. Giner-Lamia J. Cantalapiedra C. P. Botas J. Deng Z. Hernández-Plaza A.. (2024). Functional and evolutionary significance of unknown genes from uncultivated taxa. Nature 626, 377–384. doi: 10.1038/s41586-023-06955-z, PMID: 38109938 PMC10849945

[B90] Salter S. J. Cox M. J. Turek E. M. Calus S. T. Cookson W. O. Moffatt M. F.. (2014). Reagent and laboratory contamination can critically impact sequence-based microbiome analyses. BMC Biol. 12, 1–12. doi: 10.1186/s12915-014-0087-z, PMID: 25387460 PMC4228153

[B91] Santiago A. Pozuelo M. Poca M. Gely C. Nieto J. C. (2016). Alteration of the serum microbiome composition in cirrhotic patients with ascites. Sci. Rep. 6 (1), 25001. doi: 10.1038/srep25001, PMID: 27112233 PMC4845009

[B92] Scarsella E. Meineri G. Sandri M. Ganz H. H. Stefanon B. (2023). Characterization of the Blood Microbiome and Comparison with the Fecal Microbiome in Healthy Dogs and Dogs with Gastrointestinal Disease veterinary sciences Characterization of the Blood Microbiome and Comparison with the Fecal Microbiome in Healthy Dogs. Animals. (2021) 11(5), 1463. doi: 10.3390/vetsci10040277, PMID: 37104432 PMC10144428

[B93] Scarsella E. Sandri M. Stefanon B. (2020). Blood microbiome : A new marker of gut microbial population in dogs? Vet. Sci. 7 (4), 198. doi: 10.3390/vetsci7040198, PMID: 33291629 PMC7761930

[B94] Scarsella E. Zecconi A. Cintio M. (2021). Characterization of microbiome on feces, blood and milk in dairy cows with different milk leucocyte pattern. doi: 10.3390/ani11051463, PMID: 34069719 PMC8160755

[B95] Schierwagen R. Alvarez-Silva C. Madsen M. S. A. Kolbe C. C. Meyer C. Thomas D.. (2019). Circulating microbiome in blood of different circulatory compartments. Gut 68, 578–580. doi: 10.1136/gutjnl-2018-316227, PMID: 29581241

[B96] Seitz V. Schaper S. Dröge A. Lenze D. Hummel M. Hennig S. (2015). A new method to prevent carry-over contaminations in two-step PCR NGS library preparations. Nucleic Acids Res. 43, e135–e135. doi: 10.1093/nar/gkv694, PMID: 26152304 PMC4787772

[B97] Selway C. A. Eisenhofer R. Weyrich L. S. (2020). Microbiome applications for pathology: challenges of low microbial biomass samples during diagnostic testing. J. Pathol. Clin. Res. 6, 97–106. doi: 10.1002/cjp2.151, PMID: 31944633 PMC7164373

[B98] Shah N. B. Allegretti A. S. Nigwekar S. U. Kalim S. Zhao S. Lelouvier B.. (2019). Blood microbiome profile in CKD: A pilot study. Clin. J. Am. Soc Nephrol. 14, 692–701. doi: 10.2215/CJN.12161018, PMID: 30962186 PMC6500932

[B99] Shen H. Rogelj S. Kieft T. L. (2006). Sensitive, real-time PCR detects low-levels of contamination by Legionella pneumophila in commercial reagents. Mol. Cell. Probes 20, 147–153. doi: 10.1016/j.mcp.2005.09.007, PMID: 16632318

[B100] Shukla V. Singh S. Verma S. Verma S. Rizvi A. A. Abbas M. (2024). Targeting the microbiome to improve human health with the approach of personalized medicine: latest aspects and current updates. Clin. Nutr. ESPEN. 63, 813–820. doi: 10.1016/j.clnesp.2024.08.005, PMID: 39178987

[B101] Simoes-Silva L. Araujo R. Pestana M. Soares-Silva I. Sampaio-Maia B. (2018). The microbiome in chronic kidney disease patients undergoing hemodialysis and peritoneal dialysis. Pharmacol. Res. 130, 143–151. doi: 10.1016/j.phrs.2018.02.011, PMID: 29444477

[B102] Simões-Silva L. Araujo R. Pestana M. Soares-Silva I. Sampaio-Maia B. (2020). Peritoneal microbiome in end-stage renal disease patients and the impact of peritoneal dialysis therapy. Microorganisms 8, 173. doi: 10.3390/microorganisms8020173, PMID: 31991821 PMC7074711

[B103] Søby J. H. Watt S. K. Vogelsang R. P. Servant F. Lelouvier B. Raskov H.. (2020). Alterations in blood microbiota after colonic cancer surgery. BJS Open 4, 1227–1237. doi: 10.1002/bjs5.50357, PMID: 33022149 PMC7709364

[B104] Suparan K. Sriwichaiin S. Chattipakorn N. Chattipakorn S. C. (2022). Human blood bacteriome: Eubiotic and dysbiotic states in health and diseases. Cells 11, 2015. doi: 10.3390/cells11132015, PMID: 35805098 PMC9265464

[B105] Tamariz J. Voynarovska K. Prinz M. Caragine T. (2006). The application of ultraviolet irradiation to exogenous sources of DNA in plasticware and water for the amplification of low copy number DNA. J. Forensic Sci. 51, 790–794. doi: 10.1111/j.1556-4029.2006.00172.x, PMID: 16882220

[B106] Tan C. C. S. Ko K. K. K. Chen H. Liu J. Loh M. Consortium S. G. K. H.. (2023). No evidence for a common blood microbiome based on a population study of. Nat. Microbiol. 8 (5), 973–985. doi: 10.1038/s41564-023-01350-w, PMID: 36997797 PMC10159858

[B107] Tedeschi G. G. Amici D. Paparelli M. (1969). Incorporation of nucleosides and amino-acids in human erythrocyte suspensions: possible relation with a diffuse infection of mycoplasms or bacteria in the L form. Nature 222, 1285–1286. doi: 10.1038/2221285a0, PMID: 5789671

[B108] Tilahun Y. Pinango J. Q. Johnson F. Lett C. Smith K. Gipson T.. (2022). Transcript and blood − microbiome analysis towards a blood diagnostic tool for goats affected by Haemonchus contortus. Sci. Rep. 12 (1), 5362. doi: 10.1038/s41598-022-08939-x, PMID: 35354850 PMC8967894

[B109] Velmurugan G. Dinakaran V. Rajendhran J. Swaminathan K. (2020). Blood microbiota and circulating microbial metabolites in diabetes and cardiovascular disease. Trends Endocrinol. Metab. 31, 835–847. doi: 10.1016/j.tem.2020.01.013, PMID: 33086076

[B110] Vientoos-Plotts A. I. Ericsson A. C. Rindt H. Grobman M. E. Graham A. Bishop K.. (2017). Dynamic changes of the respiratory microbiota and its relationship to fecal and blood microbiota in healthy young cats. PloS One 12, 1–17. doi: 10.1371/journal.pone.0173818, PMID: 28278278 PMC5344508

[B111] Vijayvargiya P. Jeraldo P. R. Thoendel M. J. Greenwood-Quaintance K. E. Esquer Garrigos Z. Sohail M. R.. (2019). Application of metagenomic shotgun sequencing to detect vector-borne pathogens in clinical blood samples. PloS One 14, e0222915. doi: 10.1371/journal.pone.0222915, PMID: 31577814 PMC6774502

[B112] Virseda-Berdices A. BroChado-Kith O. Díez C. Hontanon V. Berenguer J. González-García J.. (2022). Blood microbiome is associated with changes in portal hypertension after successful direct-acting antiviral therapy in patients with HCV-related cirrhosis. J. Antimicrob. Chemother. 77, 719–726. doi: 10.1093/jac/dkab444, PMID: 34888660

[B113] Walsh S. J. Rau L. M. (2000). Autoimmune diseases: a leading cause of death among young and middle-aged women in the United States. Am. J. Public Health 90, 1463. doi: 10.2105/ajph.90.9.1463, PMID: 10983209 PMC1447637

[B114] Wang C. (2021). Characterization of the blood and neutrophil - specific microbiomes and exploration of potential bacterial biomarkers for sepsis in surgical patients. Microbial Pathog. 152, 1343–1357. doi: 10.1002/iid3.483, PMID: 34288545 PMC8589375

[B115] Wani A. K. Roy P. Kumar V. (2022). Metagenomics and artificial intelligence in the context of human health. Infect. Genet. Evol. 100, 105267. doi: 10.1016/j.meegid.2022.105267, PMID: 35278679

[B116] Weyrich L. S. Duchene S. Soubrier J. Arriola L. Llamas B. Breen J.. (2017). Neanderthal behaviour, diet, and disease inferred from ancient DNA in dental calculus. Nature 544, 357–361. doi: 10.1038/nature21674, PMID: 28273061

[B117] Weyrich L. S. Farrer A. G. Eisenhofer R. Arriola L. A. Young J. Selway C. A.. (2019). Laboratory contamination over time during low-biomass sample analysis. Mol. Ecol. Resour. 19, 982–996. doi: 10.1111/1755-0998.13011, PMID: 30887686 PMC6850301

[B118] Whittle E. Leonard M. O. Harrison R. Gant T. W. Tonge D. P. (2019). Multi-method characterization of the human circulating microbiome. Front. Microbiol. 10. doi: 10.3389/fmicb.2018.03266, PMID: 30705670 PMC6345098

[B119] Yang D. Wang X. Zhou X. Zhao J. Yang H. Wang S.. (2021). Blood microbiota diversity determines response of advanced colorectal cancer to chemotherapy combined with adoptive T cell immunotherapy. Oncoimmunology 10, 1976953. doi: 10.1080/2162402X.2021.1976953, PMID: 34595059 PMC8477924

[B120] Yun Y. Kim H. Chang Y. Lee Y. Ryu S. Shin H.. (2019). Characterization of the blood microbiota in korean females with rosacea. Dermatology (Basel, Switzerland) 235, 255–259. doi: 10.1159/000496968, PMID: 30844814

[B121] Zaneveld J. R. McMinds R. Vega Thurber R. (2017). Stress and stability: applying the Anna Karenina principle to animal microbiomes. Nat. Microbiol. 2, 1–8. doi: 10.1038/nmicrobiol.2017.121, PMID: 28836573

[B122] Zhang Y. Zhao R. Shi D. Sun S. Ren H. Zhao H.. (2019). Characterization of the circulating microbiome in acute - on - chronic liver failure associated with hepatitis B. Liver Intl., 39(7), 1207–1216. doi: 10.1111/liv.14097, PMID: 30864226

[B123] Zhu S. Jiang Y. Xu K. Cui M. Ye W. Zhao G.. (2020). The progress of gut microbiome research related to brain disorders. J. Neuroinflamm. 17, 1–20. doi: 10.1186/s12974-020-1705-z, PMID: 31952509 PMC6969442

